# The Glasgow Prognostic Score at Diagnosis Is a Predictor of Clinical Outcome in Patients with Multiple Myeloma Undergoing Autologous Haematopoietic Stem Cell Transplantation

**DOI:** 10.3390/cancers12040921

**Published:** 2020-04-09

**Authors:** Hanno M. Witte, Bastian Bonorden, Armin Riecke, Harald Biersack, Konrad Steinestel, Hartmut Merz, Alfred C. Feller, Veronica Bernard, Sebastian Fetscher, Nikolas von Bubnoff, Niklas Gebauer

**Affiliations:** 1 Department of Haematology and Oncology, Federal Armed Forces Hospital of Ulm, Oberer Eselsberg 40, 89081 Ulm, Germany; arminriecke@bundeswehr.org; 2 Department of Haematology and Oncology, University Hospital of Schleswig-Holstein, Campus Lübeck, Ratzeburger Allee 160, 23538 Lübeck, Germany; bbonorden@web.de (B.B.); harald.biersack@klinikum-kulmbach.de (H.B.); nikolas.vonbubnoff@uksh.de (N.v.B.); niklas.gebauer@uksh.de (N.G.); 3 Institute of Pathology and Molecular Pathology, Federal Armed Forces Hospital of Ulm, Oberer Eselsberg 40, 89081 Ulm, Germany; konradsteinestel@bundeswehr.org; 4 Hämatopathologie Lübeck, Reference Centre for Lymph Node Pathology and Haematopathology, 23538 Lübeck, Germany; merz@haematopathologie-luebeck.de (H.M.); feller@haematopathologie-luebeck.de (A.C.F.); bernard@haematopathologie-luebeck.de (V.B.); 5 Department of Haematology and Oncology, Sana Hospitals Lübeck, Kronsforder Allee 71-73, 23560 Lübeck, Germany; sebastian.fetscher@sana.de

**Keywords:** inflammation-based prognostic scores, Glasgow Prognostic Score, autologous transplantation, multiple myeloma, prognosis

## Abstract

*Background:* Immunity and inflammatory response affect the tumour microenvironment and the progression of malignancies. Metabolic and inflammatory parameters and ratios of the peripheral blood correlate with outcome in cancer patients. There exist several established and validated inflammation-based scores of prognostic significances including the Glasgow Prognostic Score (GPS). *Methods:* In this retrospective, multicentre study, we investigated the prognostic capabilities of baseline GPS in patients with multiple myeloma (MM) undergoing autologous stem cell transplantation as a complementary resource for risk stratification. For GPS calculation, a C-reactive-protein (CRP) value of >10 mg/dL counts as one point and an albumin value of <35 g/L connotes another point, resulting in three different subgroups (group I: 0 points; group II: 1 point; and group III: 2 points). Patients with MM admitted to the participating institutions between January 2010 and July 2018 were screened, and established prognostic scores and ratios were assessed. Characteristics significantly associated with overall survival (OS) or progression-free survival (PFS), upon univariate analysis, were included in a Cox proportional hazards model. *Results:* Following initial assessment, we identified 224 fully evaluable patients who underwent autologous haematopoietic stem cell transplantation for multiple myeloma. A centralised review of pathology and cytogenetic reports was conducted, and a central hematopathology assessment was performed in 175 of 224 cases (78.1%). Proceeding to high-dose chemotherapy and subsequent autologous stem cell transplantation was the main inclusion criterion for all transplant-eligible patients in the study. The median age at diagnosis was 59 years (range: 35–76 years) with a median follow-up of 76 months. Multivariate analysis revealed neutrophil–platelet score (NPS) (HR = 0.528, 95% CI = 0.284–0.984) and B symptoms at primary diagnosis (HR = 1.838, 95% CI = 1.232–2.740) to be independent predictors of PFS while high-risk cytogenetic changes (HR = 2.358, 95% CI = 1.413–3.934, *p* = 0.001) could be identified as an independent predictor of OS, and GPS to be the only independent predictor of both OS and PFS (OS: HR = 2.127, 95% CI = 1.431–3.162, *p* < 0.0001 and PFS: HR = 1.405; 95% CI = 1.058–1.867, *p* = 0.019). *Conclusions:* Our data show that baseline GPS correlates with rates of relapse and refractory disease in MM patients undergoing autologous transplantation. In a multivariate analysis, these effects were proven to hold prognostic capabilities beyond and independent from established prognosticators. These results require further validation in a prospective setting.

## 1. Introduction

Multiple myeloma (MM) is a heterogeneous, haematological neoplasia, which is characterised by a monoclonal proliferation of plasma cells and associated with an inadequate production of complete or incomplete immunoglobulins [1]. These are known as paraproteins and can be detected in serum and urine. The current WHO classification regards MM as an indolent B-cell non-Hodgkin lymphoma (NHL) [2]. MM is commonly preceded by a monoclonal gammopathy of unknown significance (MGUS). Men are affected more frequently than women (number of cases per year: +2.8% for men and +0.8% for women). The median age of onset is just over 70 years.

While in many cases, the disease may be detected incidentally on the basis of abnormal laboratory results in asymptomatic patients, acute forms are recurrently encountered in clinical practice, as well, often involving renal impairment, hematopoietic insufficiency, and prominent osteolysis with consecutive fractures. 

Multiple myeloma is a systemic haematological malignancy which is highly influenced by the composition of the bone marrow microenvironment. Multiple publications revealed that inflammatory response of the bone marrow microenvironment stimulates systemic myeloma activity. 

Life expectancy has continuously improved in recent years due to the establishment of immunomodulatory drugs (IMiDs), such as lenalidomide or pomalidomide, and the fraction of proteasome inhibitors which are represented by bortezomib or carfilzomib [3,4,5,6,7,8,9]. 

The therapeutic standard for young and fit MM patients is bortezomib-based induction treatment, followed by high-dose chemotherapy and autologous hematopoietic stem cell transplantation (HSCT) [10,11,12]. Recently published results of randomised studies have emphasised the impact of integrating novel agents, such as lenalidomide, in addition to chemotherapy in treatment regimens based on autologous HSCT. These studies have also underlined the significance of lenalidomide maintenance following autologous HSCT [12]. Further, novel agents represent a therapeutic alternative for treating relapsed or refractory diseases and also for patients who are not eligible for autologous HSCT. The use of novel agents considerably improved long-term response rates after re-evaluation following the induction treatment [13]. 

The prognostic importance of the systemic inflammatory response has been established in previous studies. The systemic inflammation has an impact on the phenotype of cellular components of the tumour microenvironment and affects levels of cytokines. C-reactive-protein (CRP) levels are regulated by several proinflammatory cytokines and the activity of infiltrating immune cells [14,15]. Another biomarker that correlates with inflammatory responses is albumin. Albumin levels also positively correlate with survival in cancer patients [15]. 

Tomographic imaging and cytogenetic analyses have been crucial in identifying high-risk patients. There is growing evidence that prognostic scores based on general inflammatory activity have a major impact on clinical outcome in various malignancies. Within the context of optimal risk stratification for other malignant haematological neoplasms, such as diffuse large B-cell lymphoma (DLBCL) and Hodgkin lymphoma, or colorectal carcinomas, systemic inflammatory scores have proven helpful [16,17,18,19,20,21,22]. These risk scores incorporate metabolic and inflammatory parameters of peripheral blood since the immunological and inflammatory response has a considerable impact on the tumour microenvironment and the progress of malignant diseases and correlates with outcome in tumour patients [16]. Apart from inflammation, different staging systems and risk scores contain nutritional components oftentimes represented by albumin serum levels at initial diagnosis. 

One such inflammation as well as nutrition-based score is the Glasgow Prognostic Score (GPS) that has been compared with further established scores and ratios in this study for transplant-eligible patients with MM. The GPS differentiates three different subgroups (group I: 0 points; group II: 1 point; and group III: 2 points) by calculating one point for CRP value of >10 mg/dL and another point for serum albumin of < 5 g/L. 

The current study hypothesizes that GPS has significant impact on survival in MM patients undergoing autologous HSCT, and therefore, the GPS should be considered as a part of individual risk stratification at initial diagnosis. 

## 2. Results

### 2.1. Clinical Characteristics of the Study Group

**Figure 1:**
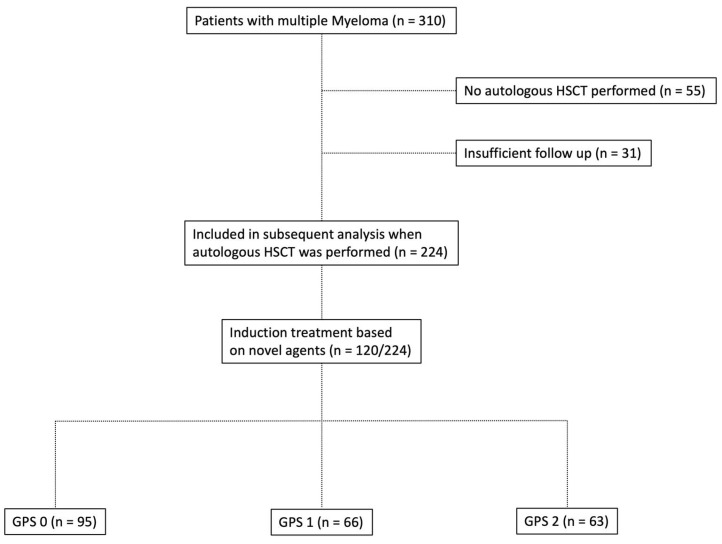
Flowchart depicting the composition of patients with multiple myeloma who underwent autologous hematopoietic stem cell transplantation (HSCT) in the study group.

Baseline characteristics of ASCT patients with MM included in the current study are briefly summarised in Table 1, whereas composition of the study group is depicted in Figure 1. The median age of onset at initial diagnosis was 59 years (range: 35–76 years). The median follow-up period was 72 months (5–260; 25% percentile 46.25; 75% percentile 122.8). A total of 130 of 224 patients included in the current study were male (58.0%). The median body mass index (BMI) was 25.35 kg/m^2^ (range: 16.85–44.75 kg/m^2^). For 140 of 224 patients (62.5%), the ECOG Performance Status was 0–2 at the time of diagnosis. A higher GPS was associated with a higher ECOG Performance Status (ECOG 0–2/3+4: Group I: 83.2%/16.8%; Group II: 69.7%/30.3%; and Group III: 57.1%/42.9%). Osteolysis was the most frequent CRAB (hypercalcaemia >2.75 mM, creatinine ≥ 2 mg/dL, haemoglobin <10 g/dL, and osteolytic bone lesions) criterion identified (195/224 cases; 87.1%). The higher the GPS, the more ASCT patients with MM were allocated to the higher stages of the Salmon and Durie, ISS, and R-ISS staging systems. Dissemination of GPS subgroups dependent on R-ISS is outlined in Table S1. Myeloma subtypes were found to be equally distributed among the various GPS groups with regard to their antibody classes and/or free light chains (FLC). The relationship between composite ratios and cumulative scores and the clinical characteristics of patients undergoing autologous HSCT is outlined in Table 2. There was statistically significant correlation between a large fraction of the assessed scores/ratios and age, B symptoms, elevated serum levels of LDH, the ECOG PS, as well as both the CCI and the R-ISS. Table 3 outlines the distribution of composite ratios/scores and their component values in the MM patients undergoing autologous stem cell transplantation. Systemic inflammation was detected in the minority of patients prior to autologous HSCT to either ratios or scores (NLR > 5 (33%); NLS = 2 (11.6%); PLR > 150 (40.2%); PLS = 2 (4.5%); NPS > 0 (17.4%); CAR > 0.22 (47.8%); GPS = 2 (28.1%); PNI < 45 (31.2%), and PI = 2 (9.3%)). 

### 2.2. Cytogenetic Aberrations

In 175 of 224 cases (78.1%), a centralised pathological review including a re-evaluation of cytogenetic characteristics was performed. In total, characteristic cytogenetic aberrations were seen more often in patients with advanced-stage GPS. 17p deletion occurred most frequently (20.1%). A complex karyotype was described in only 11.1% of the cases in GPS group I (GPS 0) but in 26.9% of the cases in GPS group III (GPS 2). High-risk cytogenetic changes investigated on a routine basis were t(4;14), t(14;16), t(14;20), and the beforementioned deletion on chromosome 17p. The frequency of cytogenetic aberrations in GPS subgroups and the underlying subtypes of MM are demonstrated in Table S2. 

### 2.3. Therapeutic Characteristics and Response Rates

Treatment modalities and their impact on response rates as well as treatment-related toxicity profiles are depicted in Table 4. All patients included in the current study received induction treatment followed by high-dose chemotherapy and autologous HSCT. For induction treatment, VCD (bortezomib, cyclophosphamide, and dexamethasone) was used most frequently in 57/224 (25.4%) MM patients. The majority of ASCT patients in this study cohort received a bortezomib- and/or lenalidomide-based induction treatment regimen (120/224; 53.6%). With regard to GPS subgroups, novel agent-based induction treatment was performed in 50 patients with GPS 0 (50/95; 52.6%), 38/63 patients with GPS 1 (57.6%), and 32/63 patients with GPS 2 (50.8%). Novel agent-based induction therapy improved initial response rates and the outcome of ASCT patients with MM in all GPS subgroups significantly while the latter, still separated divergent prognostic subgroups upon univariate analysis. Upon confirmatory multivariate survival analysis, results for overall survival were bordering on statistical significance due to shrinkage of sample size in the cohort of MM patients receiving novel-agents as induction treatment (*p* = 0.140) while only performance status retained its independent prognostic impact. Severe adverse events (SAE) due to induction treatment could be detected in 63/224 (28.1%) cases (Table 4). Haematological toxicity was highly frequented in ASCT patients with MM while receiving induction therapy (27/63 cases of SAE). Median time from diagnosis to autologous HSCT was 8 months (range: 3–146 months). Regarding IMWG response criteria, overall response rate (ORR) after transplantation for MM patients receiving any type of induction treatment was 90.2% (CR + VGPR + PR). CR-rate in this cohort was 41.1% (92/224 cases). In 55/224 cases (24.6%), CR could be detected within 120 days (CR 120) after initial cytoreductive treatment. Lenalidomide was applied in 21/224 cases as maintenance therapy after autologous HSCT. 

Predominantly, MM patients received lenalidomide (57/224 cases; 25.4%) or bortezomib (76/224; 33.9%) as second-line treatment option in relapsed or refractory setting. The toxicity profile was moderate and mostly haematological in nature with only four cases of grade IV cytopenia. Grade III cytopenia was detected in 34/224 cases (15.2%). Severe infectious complications due to cytoreductive therapy occurred in 29/224 MM patients (12.9%). 

### 2.4. Prognostic Implications of Assessed Scores/Ratios on Clinical Outcome

**Figure 2:**
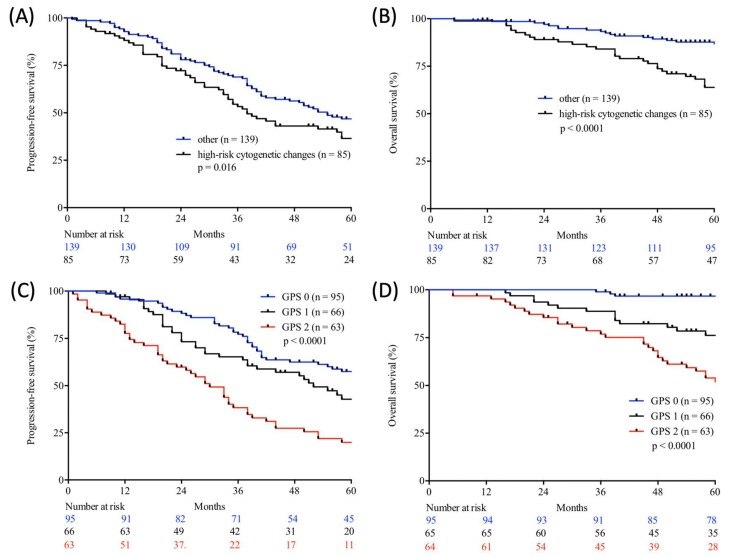
Progression-free (**A**,**C**) and overall (**B**,**D**) survival according to cytogenetic changes (log-rank test; high-risk cytogenetic changes vs. other; A, B) and Glasgow Prognostic Score (GPS) (log-rank test; GPS 0 vs. GPS 1 vs. GPS 2; C, D) in autologous stem cell transplanted (ASCT) patients with multiple myeloma (MM) from two large academic centres.

Table 5 demonstrates the correlation between the examined scores/ratios and PFS as well as OS upon initial log-rank univariate analysis. The confirmatory univariate Cox analysis is presented in Table 6. A comparative consecutive multivariate survival analysis of the total cohort of MM patients showed that high-risk cytogenetic changes (HR = 2.358, 95% CI = 1.413–3.934, *p* = 0.001) and GPS had a significant influence on OS in ASCT MM patients. For PFS, the GPS as well as the NPS (HR = 0.528, 95% CI = 0.284–0.984, *p* = 0.044) and B symptoms (HR = 1.838, 95% CI = 1.232–2.740, *p* = 0.003) at initial diagnosis were found to be independent predictors in the multivariate analysis. The influence of high-risk cytogenetic changes and GPS on OS and PFS (OS: HR = 2.127, 95% CI = 1.431–3.162, *p* < 0.0001; PFS: HR = 1.405; 95% CI = 1.058–1.867, *p* = 0.019) is demonstrated by Kaplan–Meier analysis in Figure 2. Recognising GPS subgroups as categorical variables, we continued by separately comparing clinical outcome between GPS subgroups employing the log-rank test (0 vs. 1; OS: *p* = 0.0004; HR 2.811; 95%CI 1.665–5.800; PFS: *p* = 0.0811; HR 1.449; 95% CI 0.9552–2.199; 1 vs. 2; OS: *p* = 0.0003; HR 2.395; 95%CI 1.493–3.841; PFS: *p* = 0.0006; HR 2.080; 95%CI 1.366–3.168). In addition, our dataset revealed CRP and albumin as individual components of GPS to have significant impact on both OS (*p* < 0.0001; *p* < 0.0001) and PFS (*p* < 0.0001; *p* = 0.001). These results are in keeping with results from previously published studies [23]. Our dataset, derived from multivariate Cox proportional hazard modelling including visualisation by using Forest-plots is outlined in Table 7 and Table 8. In the univariate analysis, the dichotomisation of NLS, PI, ECOG-PS >2, CCI > 3, and R-ISS and elevated serum levels of LDH >240 U/L showed an effect on MM patients’ outcome which could not be confirmed in the subsequent multivariate analysis. PFS after transplantation among 142 patients experiencing relapsed or refractory disease was 26 months (0–225 months). Median post-transplant survival was 58 months (1–248 months). In total, 90 myeloma-associated deaths were recorded during the follow-up period (40.2%).

## 3. Discussion

Our present study is the first to evaluate the prognostic qualities of inflammatory-based scores in a total of 224 patients with multiple myeloma who underwent autologous stem cell transplantation. The prognostic capabilities of the ratios and scores, considered in this study, have already been investigated and proven across a variety of malignant and other diseases [17,18]. This includes solid tumours, such as cervical cancer, hepatobiliary cancer, and non-small cell lung cancer [24,25,26,27]. Especially in patients with haematological malignancies such as diffuse large B-cell lymphoma, NK-cell/T-cell lymphoma, and Hodgkin lymphoma, the GPS was identified as an independent predictor of PFS and OS [19,21,28]. There is growing evidence, supporting the notion that the GPS, in its synopsis of synthetic liver function and acute phase reaction, constitutes one of the leading, yet widely available tools for assessing systemic inflammation of prognostic relevance in numerous malignancies. Moreover, several studies demonstrated its prognostic superiority over scores and ratios, derived from white blood cell counts, reflecting a patient’s lymphoid and/or myeloid response. In addition, Dolan et al. recently deduced a conceptual advantage for score-based approaches in comparison with inflammatory ratios due to the recurrent false-positive classification of systemically inflamed patients by the latter [20].

Previous studies have shown that NLR is an independent prognosticator of PFS and OS for gastric cancer and others [29]. Another study, however, found no correlation between NLR and PFS or OS for gastric cancer [30,31,32,33]. For this reason, the value of inflammation-based scores is a matter of some controversy, despite the general consensus that the inflammatory and immunological component in tumour tissue is becoming more significant, even from a therapeutic perspective [34]. In the present study, there was no significant prognostic impact on survival for NLR.

This is the first study to examine the prognostic value of GPS for multiple myeloma patients at diagnosis prior to autologous HSCT. 

PLR, PI, and PNI are inflammation-based scores and ratios that are also widely debated. Their independence with respect to their prognostic value for PFS or OS has not been proven in the current study. Studies on colorectal and ovarian cancer, however, have been able to illustrate the significant prognostic value of PLR [35,36]. 

We were able to demonstrate that GPS, of all the ratios and scores examined (NLR, NLS, NPS, PLR, PLS, PNI, PI, and CAR), has the highest prognostic impact on PFS and OS in our study. GPS positively correlates with age, B symptoms at initial diagnosis, the ECOG PS, an elevation of LDH serum levels, as well as with CCI and R-ISS (Table 2). 

Besides the prognostic impact of GPS, the score can be assessed very quickly. The present data could demonstrate the robust prognostic value of GPS in transplant-eligible MM patients. However, concurrent infections can lead to be diagnostically less conclusive. For a valid GPS calculation, each MM patient included in the study was screened for the possible infections that may sophisticate scoring results, and an alternative measurement time point would be determined if an infection was detected in a MM patient within the 30-days period for inclusion. 

When compared to the established R-ISS, there is superiority for GPS regarding cost effectiveness. Calculating the R-ISS, cytogenetic diagnostics are required which leads to a certain latency, whereas the components of the GPS are invariably available in the scope of initial blood sampling. Furthermore, collected biomarkers for calculation of R-ISS are less representative to reflect general inflammatory activity. 

Possible limitations of this study include its limited sample size and retrospective design, resulting in the lack of centralised pathology, laboratory, and radiology review for a subset of patients, and the potential for fragmentary data. 

Personalised risk stratification with a resulting adaptation of treatment intensity should be established in prospective trials. The majority of deaths were related to progressive disease of MM. For a small number of cases, however, due to insufficient follow-up, the cause of death remains unknown. A further limitation of this study is the possibility of selection bias, which could not be ruled out on account of the study design. Although survival of nontransplant-eligible patients with MM improved significantly due to the implementation of novel agents, the creation of a distinct definition for conditions of participation by excluding nontransplant-eligible MM patients in the current study should reduce selection bias in regard of comparability and statistical reproducibility. Moreover, due to prolongation of diagnosing multiple myeloma, a lead-time bias of maximum 30 days, especially in MM patients with progressive disease associated with end-organ damage, cannot be excluded conclusively. 

Our data show that baseline GPS correlates closely with rates of relapse and refractory disease in ASCT patients with MM treated with both novel agent-based induction regimens as well as in patients receiving conventional cytoreductive chemotherapy-based induction such as the VAD protocol. Upon multivariate analysis, these effects were preserved independent from and complementary to all other established prognosticators in multiple myeloma, including the R-ISS as the current gold standard [37]. GPS, therefore, constitutes a promising means of risk-stratification in ASCT patients with MM but requires further and preferably prospective validation, before suggestions regarding personalised risk assessment and ultimately treatment guidance can be made. 

## 4. Methods

In this retrospective, multicentre study, we investigated the prognostic value of GPS at diagnosis in autologous stem cell-transplanted (ASCT) MM patients as a complementary resource for risk stratification. All transplant-eligible MM patients from the haematology and oncology departments of University Hospital Schleswig-Holstein (Campus Lübeck) and Sana Hospital Lübeck undergoing therapy between January 2010 and July 2018 were screened with regard to their inclusion in the current study. Patients with insufficient follow-up (21 patients referred to other centres after primary diagnosis and 10 patients with subsequent loss of follow-up) or patients who did not proceed to autologous HSCT (*n* = 55) were excluded. A total of 224 MM patients undergoing autologous HSCT could be identified for whom data on prognostic factors and parameters were collected (Figure 1). For minimising lead-time bias, cases in which diagnosis of multiple myeloma after initial clinical presentation and laboratory assessment took more than 30 days were excluded. Each MM patient was screened for infections that may lead to calculation bias at initial diagnosis. An alternative measurement time point was determined within the inclusion period of 30 days when infections were detected. 

Staging was carried out using the traditional Salmon and Durie staging system and in accordance with the criteria of the Revised International Staging System (R-ISS) and also the International Staging System (ISS) of the International Myeloma Working Group (IMWG) [37]. 

### 4.1. Patient Characteristics

Clinical information was collected from the original electronic patient files. The collected data included the ECOG (Eastern Cooperative Oncology Group) Performance Status, staging data, treatment modalities, therapeutic response, pattern of relapse, survival data and the Charlson Comorbidity Index (CCI), as well as the Hematopoietic Cell Transplantation-specific Comorbidity Index (HCT-CI). The therapeutic response was evaluated according to IMWG criteria [38]. 

The laboratory data included parameters of the baseline differential blood count, serum levels of lactate dehydrogenase (LDH), myeloma-specific CRAB criteria (hypercalcaemia >2.75 mM, creatinine ≥ 2 mg/dL, haemoglobin <10 g/dL, and osteolytic bone lesions), and the detection of paraproteins (Kappa/Lambda chains and antibody classes). 

Centralised review of pathology reports was conducted and central haematopathology assessment was performed in 175/224 (78.1%) cases. In those cases, initial diagnosis was confirmed by at least two independent investigators (HM and ACF) in accordance with the WHO recommendations on formalin-fixed and paraffin-embedded (FFPE) tissue samples. Consecutively, fluorescence in situ hybridisation (FISH) was performed in order to detect cytogenetic changes which are associated with poor prognosis. Examination of high-risk cytogenetic aberrations, described by Sonneveld et al., included deletion on chromosome 17p and the translocations t(4;14), t(14;16), as well as t(14;20) [37,39,40,41]. 

### 4.2. Prognostic Scoring Systems

In this study, established prognostic scores that are based on ratios involving white blood cells were evaluated. These included the widely accepted neutrophil-to-lymphocyte ratio (NLR) and the platelet-to-lymphocyte ratio (PLR) [18,20,31,35,36,42,43]. Additionally, the neutrophil–lymphocyte score (NLS) as well as the platelet–lymphocyte score (PLS) were assessed. Another cell-count-based score which was investigated was the neutrophil–platelet score (NPS) [44].

Further prognostic scores take into account the acute-phase C-reactive protein (CRP) in serum and albumin levels as a marker of the patient’s nutritional status (CAR) [45]. Another score that includes the nutritional status is the Prognostic Nutritional Index (PNI), which is based on albumin level and total lymphocyte count [20]. The Prognostic Index (PI) regards the total white blood cell count (>10 × 10^9^/L) and the CRP value (>10 mg/dL) at initial diagnosis [32]. 

Finally, we assessed the aforementioned Glasgow Prognostic Score (GPS). A CRP value of >10 mg/dL connotes one point and an albumin value of <35 g/L counts as another point, resulting in three different subgroups (group I: 0 points; group II: 1 point; and group III: 2 points). All prognostic scores and ratios are shown in Table 9. 

### 4.3. Treatment and Assessment

Following baseline staging investigations according to standard procedures, patients were treated with a chemotherapy regimen of the treating physician’s choice with current DSMM (Deutsche Studiengruppe Multiples Myelom) study protocols serving as an institutional standard where applicable. Bone marrow aspirates and trephine biopsies were performed at initial diagnosis. PET scans were not employed on a routine basis. GPS was calculated as described above [19]. Treatment response was rated in accordance with the established criteria of complete remission (CR), very good partial remission (VGPR), and partial remission (PR). Standard definitions of overall survival (OS) and progression-free survival (PFS) were employed. In addition, toxicity profile based on National Cancer Institute Common Toxicity Criteria (NCI CTC; version 2.0) was assessed. 

### 4.4. Ethics Statement

This retrospective study was approved by the ethics committee of the University of Lübeck (reference no. 18-037) and conducted in accordance with the Declaration of Helsinki. Patients gave written informed consent regarding routine diagnostic and academic assessment of their biopsy specimen as well as transfer of their clinical data. 

### 4.5. Statistics

All statistical analyses were conducted using GraphPad PRISM 6 (GraphPad Software Inc., San Diego, CA, USA) and SPSS 26 (IBM, Armonk, NY, USA). Normality of distribution was assessed, employing the Kolmogorov–Smirnov test. Progression-free survival (PFS) and overall survival (OS) were calculated from the date of the initial diagnosis. Survival (PFS and OS) was primarily estimated by means of the Kaplan–Meier method and the univariate log-rank test. A confirmatory univariate Cox analysis was subsequently performed. Characteristics found to be associated with either OS or PFS with at least a trend towards statistical significance (*p* < 0.07) in both univariate approaches were included in a subsequent multivariate proportional hazard model (Cox proportional hazard). Differences between patient subgroups were assessed using the Chi-squared test and the Mann–Whitney *U* test as appropriate. Primarily anticipating predominantly nonlinear relationships between variables, we employed Pearson’s correlation analysis.

## 5. Conclusions

In summary, our results single out the GPS as a most promising and readily available systemic inflammation-derived prognosticator in MM patients prior to autologous HSCT with capabilities, independent from all established means of risk stratification in this population, warranting further, prospective validation in this and other therapeutic settings in MM.

## Figures and Tables

**Table 1:** Baseline characteristics for all patients included in the study.

GPS	Overall Study Group (*n* = 224)	Group I GPS 0 (*n* = 95)	Group II GPS 1 (*n* = 66)	Group III GPS 2 (*n* = 63)
Male/female	130 (58.0%)/94 (42.0%)	52 (54.7%)/43 (45.3%)	43 (65.2%)/23 (34.8%)	36 (57.1%)/27 (42.9%)
Median age (range), years	59 (35–76)	62 (35–76)	59 (39–72)	57 (38–74)
BMI (median, range)	25.35 (16.85–44.75)	24.98 (17.04–37.72)	25.39 (19.12–42.97)	25.40 (16.85–44.75)
ECOG PS				
0–2	140 (62.5%)	79 (83.2%)	46 (69.7%)	36 (57.1%)
3 + 4	84 (37.5%)	16 (16.8%)	20 (30.3%)	27 (42.9%)
CCI (median, range)	5 (2–12)	4 (2–8)	4 (2–10)	6 (2–12)
HCT-CI (median, range)	2 (0–9)	2 (0–9)	2 (0–9)	2 (0–7)
CRAB criteria				
- Calcium elev.	31	8	10	13
- Renal failure	53	11	19	23
- Anaemia	61	12	19	30
- Bone lesions	195	83	58	54
LDH level				
<240 U/L	142 (63.4%)	78 (82.1%)	38 (57.6%)	26 (41.3%)
>240 U/L	82 (36.6%)	17 (17.9%)	28 (42.4%)	37 (58.7%)
Albumin (g/L) (median, range)	37.9 (15.0–51.7)	40.6 (35.1–51.7)	30.9 (18.7–47.2)	28.1 (15.0–34.1)
>35 g/L	127 (56.7%)	95 (100.0%)	32 (48.5%)	–
<35 g/L	97 (43.3%)	–	34 (51.5%)	63 (100%)
CRP (mg/dL) (median, range)	7.9 (0.1–377.0)	3.7 (0.1–9.9)	19.6 (0.4–243.0)	67.3 (11.2–377.0)
<10 mg/dL	134 (59.8%)	95 (100.0%)	39 (59.1%)	–
>10 mg/dL	90 (40.2%)	–	27 (40.9%)	63 (100%)
Durie–Salmon stage (at diagnosis)				
I + II	77 (34.4%)	34 (35.8%)	24 (36.4%)	19 (30.2%)
III	147 (65.6%)	61 (64.2%)	42 (63.6%)	44 (69.8%)
ISS (at diagnosis)				
I	121 (54.0%)	68 (71.6%)	30 (45.5%)	23 (36.5%)
II	63 (28.1%)	21 (22.1%)	19 (28.8%)	23 (36.5%)
III	40 (17.9%)	6 (6.3%)	17 (25.7%)	17 (27.0%)
R-ISS (at diagnosis)				
I	78 (34.8%)	44 (46.3%)	20 (30.3%)	14 (22.2%)
II	115 (51.4%)	48 (50.5%)	34 (51.5%)	33 (52.4%)
III	31 (13.8%)	3 (3.2%)	12 (18.2%)	16 (25.4%)
Monoclonal component				
IgG	142 (63.4%)	61 (64.2%)	39 (59.1%)	42 (66.7%)
IgA	40 (17.9%)	20 (21.1%)	9 (13.6%)	11 (17.5%)
IgD/IgE	1 (0.4%)	–	–	1 (1.6%)
FLC only	41 (18.3%)	14 (14.7%)	18 (27.3%)	9 (14.2%)
FLC subtype				
- Kappa	149 (66.5%)	66 (69.5%)	46 (69.7%)	37 (58.7%)
- Lambda	75 (33.5%)	29 (30.5%)	20 (30.3%)	26 (41.3%)
High-risk cytogenetic changes *	85 (37.9%)	30 (31.6%)	24 (36.4%)	31 (49.2%)
- 17p-del	45 (20.1%)	15 (15.8%)	15 (22.7%)	15 (23.8%)
- t(4;14)	33 (14.7%)	13 (13.7%)	8 (12.1%)	12 (19.0%)
- t(14;16)	27 (10.7%)	10 (10.5%)	5 (7.6%)	12 (19.0%)
- t(14;20)	19 (8.5%)	3 (3.1%)	6 (9.1%)	10 (15.9%)
- CKt	38 (16.9%)	11 (11.6%)	8 (12.1%)	17 (26.9%)

CCI, Charlson Comorbidity Index; ECOG PS, Eastern Cooperative Oncology Group Performance Status; FLC, free light chain; ISS, International Staging System; R, revised, R-ISS, Revised International Staging System; * High-risk cytogenetic changes include t(4;14), t(14;16), t(14;20), deletion 17p, and complex karyotype abnormalities (CKt).

**Table 2:** Pearson’s correlation between composite ratios and cumulative scores and baseline clinicopathological characteristics of patients with multiple myeloma undergoing autologous hematopoietic stem cell transplantation (HSCT) (*n* = 224).

	Age	Sex	BMI	B Symptoms	ECOG	LDH	CCI	S&D	R-ISS
NLR	0.654	0.354	0.499	**0.047**	0.164	**<0.0001**	0.2265	0.535	0.135
NLS	0.951	0.566	0.836	**0.023**	0.077	**0.0006**	**0.001**	0.115	0.223
PLR	0.269	0.183	0.611	0.401	0.638	0.79	0.443	0.731	0.370
PLS	0.837	0.438	0.405	0.101	0.361	0.079	**0.044**	0.145	0.517
NPS	0.951	0.531	0.853	**<0.0001**	0.051	**<0.0001**	0.067	0.879	**0.018**
CAR	0.195	0.685	0.335	**<0.0001**	**<0.0001**	**<0.0001**	**<0.0001**	0.054	**0.0003**
PI	0.005	0.857	0.246	**<0.0001**	**<0.0001**	**<0.0001**	**<0.0001**	**0.038**	**<0.0001**
PNI	0.119	0.951	0.876	**0.002**	**<0.0001**	**<0.0001**	**<0.0001**	0.856	0.0002
HR-CC	0.626	0.051	0.386	**0.008**	**0.035**	**0.009**	0.164	**0.016**	**<0.0001**
GPS	0.015	0.594	0.17	**0.0004**	**<0.0001**	**<0.0001**	**<0.0001**	0.097	**<0.0001**

* *p* < 0.05 is considered significant. Significant results are highlighted in bold. BMI, body mass index; CAR, C-reactive protein albumin ratio; HR-CC, high-risk cytogenetic changes; CCI, Charlson Comorbidity Index; ECOG, Eastern Cooperative Oncology Group; GPS, Glasgow Prognostic Score; ISS, International Staging System; LDH, lactate-dehydrogenase; NLR, neutrophil–lymphocyte ratio; NLS, neutrophil–lymphocyte score; NPS, neutrophil–platelet score; PI, Prognostic Index; PLR, platelet–lymphocyte ratio; PLS, platelet–lymphocyte score; R-ISS, Revised International Staging System; S&D, Salmon and Durie.

**Table 3:** The relationship between composite ratios and cumulative scores and their component values in autologous stem cell transplanted (ASCT) patients with multiple myeloma (MM) (*n* = 224).

Scores/Ratios	*n* (%)	Median (Range) Neutrophil (×10^9^/L)	Median (Range) Lymphocyte (×10^9^/L)
NLR			
≤ 3	100 (44.6 %)	5.97 (0.81–13.49)	0.55 (0.03–1.40)
3–5	50 (22.3 %)	3.95 (0.20–8.21)	1.10 (0.05–2.71)
> 5	74 (33.0 %)	2.34 (0.46–9.22)	1.50 (0.22–5.45)
NLS			
0	65 (29.0 %)	4.37 (1.40–7.35)	1.95 (1.50–5.45)
1	133 (59.4 %)	2.82 (0.20–9.22)	2.31 (0.03–4.58)
2	26 (11.6 %)	9.75 (7.53–13.47)	0.66 (0.12–1.40)
		Platelet (×10^9^/L)	Lymphocyte (×10^9^/L)
PLR			
≤150	134 (59.8 %)	216 (43–662)	0.73 (0.03–2.30)
>150	90 (40.2 %)	150 (24–455)	1.63 (0.29–5.45)
PLS			
0	65 (29.0 %)	208 (70–394)	2.00 (1.50–4.96)
1	149 (66.5 %)	163 (24–662)	0.74 (0.03–5.45)
2	10 (4.5 %)	442 (410–662)	0.63 (0.16–0.95)
		Neutrophil (×10^9^/L)	Platelet (×10^9^/L)
NPS			
0	185 (82.6 %)	3.25 (0.20–7.35)	187 (24–394)
1	38 (17.0 %)	8.81 (0.46–13.49)	173.5 (43–662)
2	1 (0.4 %)	13.75 (-)	662 (-)
		Albumin (g/L)	CRP (mg/dL)
CAR			
≤0.22	117 (52.2 %)	40.5 (19.9–59.0)	2.4 (0.1–10.1)
>0.22	107 (47.8 %)	30.8 (15.0–58.4)	22.8 (7.6–377.0)
GPS			
0	95 (42.4 %)	41.6 (34.8–59.0)	2.5 (0.1–41.0)
1	66 (29.5 %)	35.1 (17.3–58.4)	9.6 (0.4–210.0)
2	63 (28.1 %)	27.6 (15.0–34.5)	38.3 (11.2–377.0)
		Albumin (g/L)	Lymphocyte (×10^9^/L)
PNI			
≥45	154 68.8 %)	32.7 (15.0 – 44.9)	0.75 (0.03 – 3.60)
<45	70 (31.2 %)	42.8 (24.1 – 59.0)	1.61 (0.27 – 5.45)
		WBC (×10^9^/L)	CRP (mg/dL)
PI			
0	122 (54.5 %)	4.89 (0.96–9.20)	2.9 (0.1–9.9)
1	81 (36.2 %)	3.51 (0.25–12.06)	19.9 (1.4–322.0)
2	21 (9.3 %)	11.57 (11.0–13.91)	41.0 (11.2–377.0)

NLR neutrophil–lymphocyte ratio, NLS neutrophil–lymphocyte score, CAR C-reactive protein albumin ratio, CRP C-reactive protein, GPS Glasgow Prognostic Score, NPS neutrophil–platelet score, PI Prognostic Index, PLR platelet–lymphocyte ratio, PLS platelet–lymphocyte score, PNI Prognostic Nutritional Index, WBC white blood cell count.

**Table 4:** Therapeutic characteristics of all patients included in the study.

GPS	Overall Study Group (*n* = 224)	Group I GPS 0 (*n* = 95)	Group II GPS 1 (*n* = 66)	Group III GPS 2 (*n* = 63)
Induction				
- VRD	17 (7.6%)	6 (6.3%)	7 (10.6%)	4 (6.3%)
- VCD	57 (25.4%)	32 (33.7%)	12 (18.2%)	13 (20.6%)
- VAD	44 (19.6%)	25 (26.3%)	15 (22.7%)	4 (6.3%)
- VD	46 (20.5%)	12 (12.6%)	19 (28.8%)	15 (23.8%)
- Other treatment	60 (26.8%)	20 (21.1%)	13 (19.7%)	27 (42.9%)
- SAE (toxicity)	63 (28.1%)	22 (23.2%)	22 (33.3%)	19 (30.2%)
Mobilisation				
- Cyclophosphamide	102 (45.5%)	39 (41.0%)	32 (48.5%)	33 (52.4%)
- Melphalan	17 (7.6%)	3 (3.2%)	6 (9.1%)	8 (12.7%)
- CAD	70 (31.3%)	46 (48.4%)	22 (33.3%)	4 (6.3%)
- IEV	33 (14.7%)	7 (7.4%)	6 (9.1%)	18 (28.6%)
Median time from diagnosis to auto-HSCT (range), months	8 (3–146)	7 (3–146)	8 (3–90)	7 (3–109)
Best response after transplantation (IMWG)				
CR	92 (41.1%)	39 (41.1%)	32 (48.5%)	21 (33.3%)
VGPR	52 (23.2%)	22 (23.2%)	14 (21.2%)	16 (25.4%)
PR	58 (25.9%)	27 (28.4%)	14 (21.2%)	17 (27.0%)
SD	10 (4.5%)	5 (5.2%)	2 (3.0%)	3 (4.8%)
PD	12 (5.4%)	2 (2.1%)	4 (6.1%)	6 (9.5%)
CR120	55 (24.6%)	29 (30.5%)	19 (28.8%)	7 (11.1%)
Maintenance				
Lenalidomide	21 (9.4%)	10 (10.5%)	6 (9.1%)	5 (7.9%)
Second-line therapy				
Lenalidomide	57 (25.4%)	19 (20.0%)	21 (31.8%)	17 (27.0%)
Bortezomib	76 (33.9%)	31 (32.6%)	19 (28.8%)	26 (41.3%)
Thalidomide	25 (11.2%)	12 (12.6%)	3 (4.5%)	10 (15.8%)
Pomalidomide	6 (2.7%)	1 (1.1%)	4 (6.1%)	1 (1.6%)
Elotuzumab	3 (1.3%)	2 (2.1%)	–	1 (1.6%)
Daratumumab	3 (1.3%)	–	1 (1.5%)	2 (3.2%)
Carfilzomib	6 (2.7%)	1 (1.1%)	2 (3.0%)	3 (4.8%)
Toxicity				
Cytopenia grade III/IV	38 (17.0%)	10 (10.55%)	9 (13.6%)	19 (30.2%)
Pneumonia	18 (8.0%)	7 (7.4%)	4 (6.1%)	7 (11.1%)
Sepsis	11 (4.9%)	3 (3.2%)	4 (6.1%)	4 (6.3%)
Neuropathy	11 (4.9%)	4 (4.2%)	2 (3.0%)	5 (7.9%)
Cardiotoxicity	8 (3.6%)	3 (3.2%)	1 (1.5%)	4 (6.3%)

CAD, cyclophosphamide/doxorubicin/dexamethasone; CR, complete remission; CR120, complete response within 120 days after initial treatment; IEV, ifosfamide/epirubicin/etoposide; IMWG, International Myeloma Working Group; PR, partial remission; PD, progressive disease; SAE, severe adverse events; SD, stable disease; VAD, vincristine/doxorubicin/dexamethasone; VCD, bortezomib/cyclophosphamide/dexamethasone; VD, bortezomib/dexamethasone; VRD, bortezomib/lenalidomide/dexamethasone; VGPR, very good partial remission.

**Table 5:** Progression-free and overall survival in univariate analysis (log-rank test).

Univariate Analysis		
Prognostic Factor	*p* Value	
	PFS	OS
GPS	**<0.0001**	**<0.0001**
C-reactive-protein	**<0.0001**	**<0.0001**
Albumin	**0.001**	**<0.0001**
Cytogenetics	**0.016**	**<0.0001**
NLR	0.143	0.077
NLS	0.151	**0.012**
PLR	0.566	0.828
PLS	0.176	0.127
NPS	0.396	0.022
CAR	0.089	0.106
PI	**<0.0001**	**<0.0001**
Age > 65 years	0.193	0.324
B symptoms	**0.001**	**0.012**
ECOG PS > 2	**<0.0001**	**<0.0001**
Elevated LDH	**0.003**	**<0.0001**
CCI > 3	**0.013**	**<0.0001**
Salmon and Durie I/II vs. III	0.568	0.269
R-ISS	0.081	**<0.0001**

CAR, C-reactive-protein albumin ratio; CCI, Charlson Comorbidity Index; ECOG PS, Eastern Cooperative Oncology Group Performance Status; GPS, Glasgow Prognostic Score; HR, hazard ratio; LDH, lactate dehydrogenase; NLR, neutrophil-to-lymphocyte ratio; NLS, neutrophil–lymphocyte score; NPS, neutrophil–platelet score; OS, overall survival; PFS, progression-free survival; PI, Prognostic Index; PLR, platelet-to-lymphocyte ratio; PLS, platelet–lymphocyte score; R-ISS, Revised International Staging System. Bold values indicate statistical significance (*p* < 0·05) in univariate log-rank test.

**Table 6:** Progression-free and overall survival in univariate analysis (univariate Cox analysis).

Univariate Analysis				
Prognostic Factor	PFS		OS	
	*p* Value	HR (95% CI)	*p* Value	HR (95% CI)
GPS	**<0.0001**	1.702 (1.397–2.073)	**<0.0001**	2.604 (1.999–3.391)
CRP	**<0.0001**	2.111 (1.531–2.911)	**<0.0001**	3.699 (2.404–5.691)
Albumin	**0.001**	1.747 (1.262–2.419)	**<0.0001**	3.130 (2.041–4.799)
Cytogenetics	**0.017**	1.484 (1.073–2.054)	**<0.0001**	2.865 (1.877–4.373)
NLR	0.145	1.148 (0.953–1.383)	**0.042**	1.298 (1.012–1.665)
NLS	0.078	1.279 (0.973–1.682)	**0.004**	1.755 (1.201–2.567)
PLR	0.569	1.098 (0.795–1.518)	0.829	0.955 (0.628–1.451)
PLS	0.142	1.250 (0.928–1.683)	0.082	1.416 (0.955–2.100)
NPS	0.936	1.018 (0.661–1.566)	0.133	1.521 (0.903–2.562)
CAR	**<0.0001**	2.133 (1.545–2.945)	**<0.0001**	4.084 (2.601–6.412)
PI	**<0.0001**	1.591 (1.269–1.994)	**<0.0001**	2.350 (1.746–3.163)
Age < 65 years	0.197	1.274 (0.882–1.841)	0.315	0.772 (0.460–1.295)
B symptoms	**0.001**	1.839 (1.280–2.642)	**0.018**	1.761 (1.124–2.757)
ECOG PS > 2	**<0.0001**	1.848 (1.334–2.560)	**<0.0001**	2.692 (1.773–4.087)
Elevated LDH	**0.004**	1.630 (1.169–2.272)	**<0.0001**	2.313 (1.522–3.519)
CCI > 3	**0.015**	1.667 (1.106–2.513)	**<0.0001**	2.934 (1.587–5.424)
Salmon and Durie I/II vs. III	0.571	1.103 (0.785–1.550)	0.263	1.292 (0.819–2.039)
R-ISS	**0.043**	1.288 (1.009–1.645)	**<0.0001**	2.075 (1.482–2.905)

CAR, C-reactive-protein albumin ratio; CCI, Charlson Comorbidity Index; CRP, C-reactive protein; ECOG PS, Eastern Cooperative Oncology Group Performance Status; GPS, Glasgow Prognostic Score; HR, hazard ratio; LDH, lactate dehydrogenase; NLR, neutrophil-to-lymphocyte ratio; NLS, neutrophil–lymphocyte score; NPS, neutrophil–platelet score; OS, overall survival; PFS, progression-free survival; PI, Prognostic index; PLR, platelet-to-lymphocyte ratio; PLS, platelet–lymphocyte score; R-ISS, Revised International Staging System. Bold values indicate statistical significance (*p* < 0·05) in univariate Cox analysis.

**Table 7:**
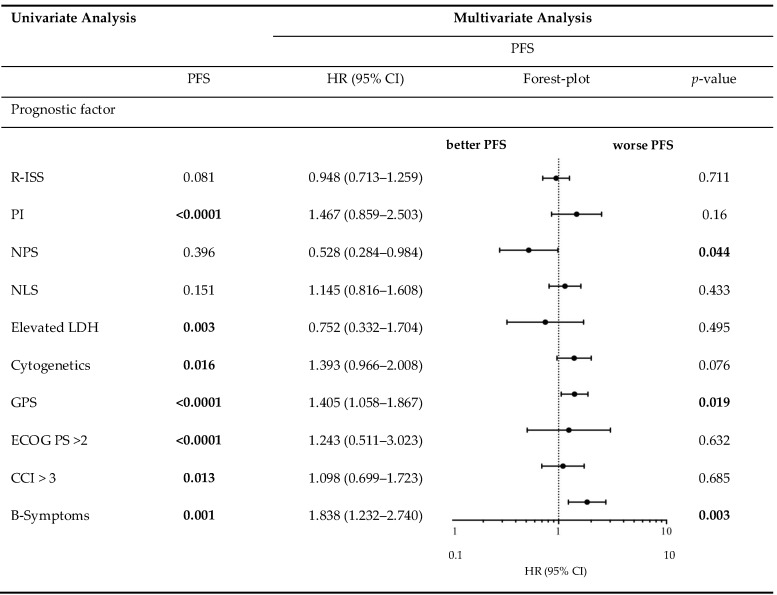
Progression-free survival in univariate analysis and consecutive multivariate Cox proportional hazard regression.

CCI, Charlson Comorbidity Index; ECOG PS, Eastern Cooperative Oncology Group performance status; GPS, Glasgow Prognostic Score; HR, Hazard ratio; LDH, lactate dehydrogenase; NLS, neutrophil-lymphocyte score; NPS, neutrophil-platelet score; OS, overall survival; PI, prognostic index; Bold values maintain their statistical significance (*p* < 0·05) in multivariate analysis.

**Table 8:**
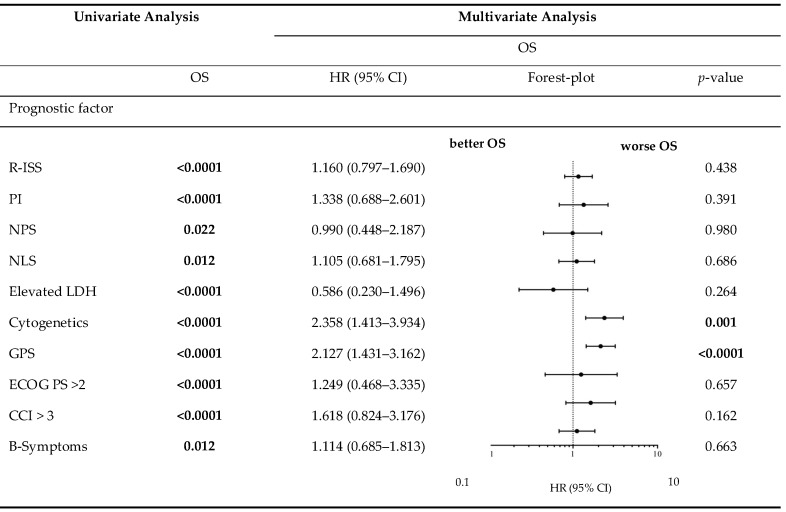
Overall survival in univariate analysis and consecutive multivariate Cox proportional hazard regression.

CCI, Charlson Comorbidity Index; ECOG PS, Eastern Cooperative Oncology Group performance status; GPS, Glasgow Prognostic Score; HR, Hazard ratio; LDH, lactate dehydrogenase; NLS, neutrophil-lymphocyte score; NPS, neutrophil-platelet score; OS, overall survival; PI, prognostic index; Bold values maintain their statistical significance (*p* < 0·05) in multivariate analysis.

**Table 9:** Systemic inflammation-based prognostic ratios and scores.

Ratio/Score	Ratio/Score
NLR	
Neutrophil count: lymphocyte count	≤3
Neutrophil count: lymphocyte count	3–5
Neutrophil count: lymphocyte count	> 5
NLS	
Neutrophil count ≤7.5 × 10^9^/L and lymphocyte count ≥1.5 × 10^9^/L	0
Neutrophil count >7.5 × 10^9^/L and lymphocyte count ≥1.5 × 10^9^/L	1
Neutrophil count ≤7.5 × 10^9^/L and lymphocyte count <1.5 × 10^9^/L	1
Neutrophil count >7.5 × 10^9^/L and lymphocyte count <1.5 × 10^9^/L	2
PLR	
Platelet count: lymphocyte count	≤150
Platelet count: lymphocyte count	>150
PLS	
Platelet count ≤400 × 10^9^/L and lymphocyte count ≥1.5 × 10^9^/L	0
Platelet count >400 × 10^9^/L and lymphocyte count ≥1.5 × 10^9^/L	1
Platelet count ≤400 × 10^9^/L and lymphocyte count <1.5 × 10^9^/L	1
Platelet count >400 × 10^9^/L and lymphocyte count <1.5 × 10^9^/L	2
PI	
White blood cell count ≤10 × 10^9^/L and C-reactive protein ≤10 mg/L	0
White blood cell count ≤10 × 10^9^/L and C-reactive protein >10 mg/L	1
White blood cell count >10 × 10^9^/L and C-reactive protein ≤10 mg/L	1
White blood cell count >10 × 10^9^/L and C-reactive protein >10 mg/L	2
PNI	
Albumin (g/L) + 5 × (lymphocyte count (10^9^/L))	≥45
Albumin (g/L) + 5 × (lymphocyte count (10^9^/L))	>45
NPS	
Neutrophil count ≤7.5 × 10^9^/L and platelet count <400 × 10^9^/L	0
Neutrophil count >7.5 × 10^9^/L and platelet count <400 × 10^9^/L	1
Neutrophil count ≤7.5 × 10^9^/L and platelet count >400 × 10^9^/L	1
Neutrophil count >7.5 × 10^9^/L and platelet count >400 × 10^9^/L	2
CAR	
C-reactive protein: albumin	≤0.22
C-reactive protein: albumin	>0.22
GPS	
C-reactive protein ≤10 mg/L and albumin ≥35 g/L	0
C-reactive protein >10 mg/L or albumin <35 g/L	1
C-reactive protein >10 mg/L and albumin <35 g/L	2

NLR, neutrophil–lymphocyte ratio; NLS, neutrophil–lymphocyte score; CAR, C-reactive protein albumin ratio; GPS, Glasgow Prognostic Score; NPS, neutrophil–platelet score; PI, Prognostic Index; PLR, platelet–lymphocyte ratio; PLS, platelet–lymphocyte score; PNI, Prognostic Nutritional Index.

## Supplementary Materials

The following are available online at https://www.mdpi.com/2072-6694/12/4/921/s1, Table S1: specific characteristics at initial diagnosis for ASCT patients with MM based on R-ISS, Table S2: cytogenetic aberrations and associated multiple myeloma subtypes in the study cohort.
