# Supplementary material for: The Glasgow Prognostic Score at Diagnosis Is a Predictor of Clinical Outcome in Patients with Multiple Myeloma Undergoing Autologous Haematopoietic Stem Cell Transplantation
Source: Cancers (Basel). 2020 Apr 9;12(4):921. doi: 10.3390/cancers12040921 (PMC7226410; doi:10.3390/cancers12040921)
Supplement: Supplementary file 1 [file cancers-12-00921-s001.pdf]

# Supplementary Materials: The Glasgow Prognostic Score at Diagnosis Is a Predictor of Clinical Outcome in Patients with Multiple Myeloma Undergoing Autologous Haematopoietic Stem Cell Transplantation

**Hanno M. Witte, Bastian Bonrorden, Armin Riecke, Harald Biersack, Konrad Steinestel, Hartmut Merz, Alfred C. Feller, Veronica Bernard, Sebastian Fetscher, Nikolas von Bubnoff and Niklas Gebauer**

**Table S1.** Specific characteristics at initial diagnosis for ASCT patients with MM based on R-ISS.

| **Characteristics** | **R-ISS I (*n* = 78)** | **R-ISS II (*n* = 115)** | **R-ISS III (*n* =31)** |
|---|---|---|---|
| **GPS** | | | |
| - GPS 0 | 44 (56.4%) | 48 (41.7%) | 3 (9.7%) |
| - GPS 1 | 20 (25.6%) | 34 (29.6%) | 12 (38.7%) |
| - GPS 2 | 14 (17.9%) | 33 (28.7%) | 16 (51.6%) |
| **High risk cytogenetic changes *** | | | |
| - 17p del | 3 (3.8%) | 31 (26.9%) | 11 (35.5%) |
| - t(4;14) | 4 (5.2%) | 21 (18.3%) | 8 (28.8%) |
| - t(14;16) | 3 (3.8%) | 15 (13.0%) | 9 (29.0%) |
| - t(14;20) | 2 (2.6%) | 11 (9.6%) | 6 (19.4%) |
| **Monoclonal component** | | | |
| - IgG | 54 (69.2%) | 70 (60.9%) | 18 (58.1%) |
| - IgA | 13 (16.7%) | 23 (20.0%) | 4 (12.9%) |
| - IgD/IgE | - | 1 (0.9%) | - |
| - FLC only | 11 (14.1%) | 21 (18.3%) | 9 (29.0%) |
| **FLC subtype** | | | |
| - Kappa | 57 (73.1%) | 74 (64.3%) | 17 (54.8%) |
| - Lambda | 21 (26.9%) | 41 (35.7%) | 14 (45.2%) |

FLC, free light chain; R-ISS, revised International Staging System; * High-risk cytogenetic changes include t(4;14), t(14;16), t(14;20) and deletion 17p

**Table S2.** Cytogenetic aberrations and associated multiple myeloma subtypes in the study cohort.

| **High risk cytogenetic changes** | **17p del** (*n* = 45) | **t (4;14)** (*n* = 33) | **t (14:16)** (*n* = 27) | **t (14;20)** (*n* = 19) | **CKt** (*n* = 38) |
|---|---|---|---|---|---|
| **GPS** | | | | | |
| - GPS 0 | 15 (33.3%) | 13 (39.4%) | 10 (37.1%) | 3 (15.8%) | 11 (28.9%) |
| - GPS 1 | 15 (33.3%) | 8 (24.2%) | 5 (18.5%) | 6 (31.6%) | 8 (21.1%) |
| - GPS 2 | 15 (33.3%) | 12 (36.4%) | 12 (44.4%) | 10 (52.6%) | 17 (44.7%) |
| **Monoclonal component** | | | | | |
| - IgG | 29 (64.4%) | 18 (54.5%) | 22 (81.5%) | 12 (63.2%) | 24 (63.2%) |
| - IgA | 7 (15.6%) | 11 (33.3%) | 4 (14.8%) | 3 (15.8%) | 8 (21.1%) |
| - IgD/IgE | - | 1 (3.0%) | - | - | - |
| - FLC only | 9 (20.0%) | 3 (9.1%) | 1 (3.7%) | 4 (21.1%) | 6 (15.8%) |
| **FLC subtype** | | | | | |
| - Kappa | 26 (57.8%) | 18 (54.5%) | 16 (59.3%) | 12 (63.2%) | 19 (50.0%) |
| - Lambda | 19 (42.2%) | 15 (45.5%) | 11 (40.7%) | 7 (36.8%) | 19 (50.0%) |

FLC, free light chain; * High-risk cytogenetic changes include t(4;14), t(14;16), t(14;20), deletion 17p and complex karyotype abnormalities (CKt)

© 2020 by the authors. Licensee MDPI, Basel, Switzerland. This article is an open access article distributed under the terms and conditions of the Creative Commons Attribution (CC BY) license (http://creativecommons.org/licenses/by/4.0/).
